# Multispectral Imaging Algorithm Predicts Breslow Thickness of Melanoma

**DOI:** 10.3390/jcm11010189

**Published:** 2021-12-30

**Authors:** Szabolcs Bozsányi, Noémi Nóra Varga, Klára Farkas, András Bánvölgyi, Kende Lőrincz, Ilze Lihacova, Alexey Lihachev, Emilija Vija Plorina, Áron Bartha, Antal Jobbágy, Enikő Kuroli, György Paragh, Péter Holló, Márta Medvecz, Norbert Kiss, Norbert M. Wikonkál

**Affiliations:** 1 Department of Dermatology, Venereology and Dermatooncology, Semmelweis University, 1085 Budapest, Hungary; bozsanyi.szabolcs@med.semmelweis-univ.hu (S.B.); varga.noemi@stud.semmelweis.hu (N.N.V.); farkas.klara@phd.semmelweis.hu (K.F.); banvolgyi.andras@med.semmelweis-univ.hu (A.B.); lorincz.kende@med.semmelweis-univ.hu (K.L.); antaljobbagy@gmail.com (A.J.); kuroli.eniko@med.semmelweis-univ.hu (E.K.); hollo.peter@med.semmelweis-univ.hu (P.H.); medvecz.marta@med.semmelweis-univ.hu (M.M.); kiss.norbert@med.semmelweis-univ.hu (N.K.); 2 Selye János Doctoral College for Advanced Studies, Clinical Sciences Research Group, 1085 Budapest, Hungary; 3 Biophotonics Laboratory, Institute of Atomic Physics and Spectroscopy, University of Latvia, 1004 Riga, Latvia; ilze.lihacova@lu.lv (I.L.); aleksejs.lihacovs@lu.lv (A.L.); emilija_vija.plorina@lu.lv (E.V.P.); 4 Department of Bioinformatics, Semmelweis University, 1085 Budapest, Hungary; bartha.aron@med.semmelweis-univ.hu; 5 2nd Department of Pediatrics, Semmelweis University, 1085 Budapest, Hungary; 6 1st Department of Pathology and Experimental Cancer Research, Semmelweis University, 1085 Budapest, Hungary; 7 Department of Dermatology, Roswell Park Comprehensive Cancer Center, Buffalo, NY 14203, USA; gyorgy.paragh@roswellpark.org; 8 Department of Cell Stress Biology, Roswell Park Comprehensive Cancer Center, Buffalo, NY 14203, USA

**Keywords:** melanoma, surgery, Breslow thickness, LED, dermoscopy, quantitative analysis, melanin, multispectral imaging, histology, diagnosis

## Abstract

Breslow thickness is a major prognostic factor for melanoma. It is based on histopathological evaluation, and thus it is not available to aid clinical decision making at the time of the initial melanoma diagnosis. In this work, we assessed the efficacy of multispectral imaging (MSI) to predict Breslow thickness and developed a classification algorithm to determine optimal safety margins of the melanoma excision. First, we excluded nevi from the analysis with a novel quantitative parameter. Parameter s’ could differentiate nevi from melanomas with a sensitivity of 89.60% and specificity of 88.11%. Following this step, we have categorized melanomas into three different subgroups based on Breslow thickness (≤1 mm, 1–2 mm and >2 mm) with a sensitivity of 78.00% and specificity of 89.00% and a substantial agreement (κ = 0.67; 95% CI, 0.58–0.76). We compared our results to the performance of dermatologists and dermatology residents who assessed dermoscopic and clinical images of these melanomas, and reached a sensitivity of 60.38% and specificity of 80.86% with a moderate agreement (κ = 0.41; 95% CI, 0.39–0.43). Based on our findings, this novel method may help predict the appropriate safety margins for curative melanoma excision.

## 1. Introduction

Melanoma is a malignant melanocytic tumor that accounts for the majority of the skin cancer-related mortality [1,2,3]. Approximately 232,100 new invasive melanomas are diagnosed worldwide, and melanoma accounts for more than 55,000 deaths annually [4]. Melanoma has four main subtypes: (1) superficial spreading melanoma (SSM), (2) nodular melanoma (NM), (3) lentigo maligna melanoma (LMM), and (4) acral lentiginous melanoma (ALM) [5]. According to the American Academy of Dermatology, the National Institute of Health, and the National Comprehensive Cancer Network, surgical excision followed by histopathological evaluation is the gold standard for diagnosing melanoma [6,7]. The Breslow tumor thickness is the maximum perpendicular invasion of the tumor, the distance in millimeters between the granular layer of the dermis or the base of ulceration, and the deepest point invaded by tumor cells [6,8] not including deeper follicular or adventitial extension [6]. The presumed or confirmed tumor depth is a vital element of the tumor staging [9], which defines the required surgical safety margin [10]. If histology finds thicker melanoma than clinically expected and the melanoma excision had insufficient surgical margins, reoperation is needed. Breslow thickness is the strongest predictor of metastatic spread [11] and determines the need for sentinel lymph node biopsy (SLNB). SLNB is required if Breslow thickness is more than 0.8 mm [12].

Non-invasive optical imaging modalities have great potential in melanoma diagnosis and the estimation of tumor depth. Dermoscopy is the most widely used skin imaging tool in dermatology. Among various other applications, it has also been applied to predict Breslow thickness [13]. Specific dermoscopic patterns can be helpful in predicting thickness. Light brown color, atypical pigment network, regression, and hypopigmented areas are typically present in thin melanomas. In contrast, thick melanomas are associated with blue-white veil, milky red areas, blue-black pigmentation, irregular vessels, shiny white streaks, rainbow pattern, ulceration, and pseudolacunae [14]. 

Various non-invasive imaging methods have been used to assess melanoma thickness, such as high-frequency ultrasound (HFUS) [15] and photoacoustic microscopy [16]. Optical coherence tomography (OCT) is a potent tool to diagnose melanoma in vivo based on the structural and visual characteristics [17] and can predict the depth of thin melanomas (<400 mm) [18]. HFUS was more suitable to measure the thickness of deeper lesions (>400 mm) [18]. Reflectance confocal microscopy (RCM) is a non-invasive imaging modality which can differentiate melanomas from other skin lesions based on their visible and characteristic patterns in vivo [19] and ex vivo [20]. RCM could classify melanomas into thick (>1 mm) and thin (≤1 mm) subgroups in vivo [21]. Another confocal microscopy modality, confocal laser scanning microscopy, proved to be a promising tool to estimate preoperative tumor thickness also, and its ex vivo results correlated with the histological findings [22]. These non-invasive imaging techniques are capable of providing a diagnosis of melanoma and estimate its depth, but their prices are high and their use requires special training and expertise. 

Multispectral imaging (MSI) is an emerging diagnostic technique [23,24] that uses different wavelength bands to capture images [25] mostly between the visible and infrared light spectrum (400–970 nm) provided by light bulbs or LED lights [26]. This method combines the advantages of spectrophotometry (spectral resolution) and digital cameras (spatial resolution) [23]. MSI has been used formerly to distant map skin chromophores, such as hemoglobin and melanin [27]. The primary advantage of MSI compared to other imaging modalities is its cost-effectiveness and that it can also be implemented into smartphone cameras [28,29]. Recently, MSI was also used to detect skin cancer recurrence [30,31]. MSI can also differentiate benign lesions from malignant tumors based on their autofluorescence intensity (AF) [32]. Our research group has previously shown this technique to differentiate seborrheic keratosis from melanoma [33]. In addition, we introduced MSI for the diagnostics of rare skin disorders, including pseudoxanthoma elasticum [34] and keratinopathic ichthyosis [35]. 

In our present study, we aimed to develop and assess a novel MSI algorithm for melanoma Breslow tumor thickness prediction, and thus the determination of optimal safety margins of melanoma surgeries. Then we compared the algorithm to the performance of clinical assessment by dermatologists and dermatology residents. 

## 2. Materials and Methods

### 2.1. Inclusion Criteria

In this study we included primary cutaneous melanomas histologically verified by expert dermatopathologists, and nevi confirmed by two expert dermatologists with clinical and dermoscopic examination. Only lesions on body parts accessible to the MSI device were investigated. If the lesion was larger than the field of view, multiple image sets were captured. 

### 2.2. Exclusion Criteria

We excluded cases where histopathological report was not available, cases that proved to be melanoma metastases, as well as primary melanomas of special sites (acral, genital, or mucosal melanoma), and melanomas with tumor thickness higher than 10 mm. Thick hair density, bleeding, or scales that impeded an adequate dermoscopic evaluation were also excluded. In situ melanomas were also excluded from this study.

### 2.3. Multispectral Imaging and Analysis of Intensity Values and Shape Descriptors

MSI was performed at the Department of Dermatology, Venereology and Dermatooncology, Semmelweis University (Budapest, Hungary) and at the Oncology Center of Latvia (Riga, Latvia). The handheld prototype used in this study was developed by the University of Latvia in collaboration with Riga Technical University (Riga, Latvia). The illumination source was an LED ring which contained four types of LED-diodes with wavelengths of 405 nm (autofluorescence/AF), 525 nm (green/G), 660 nm (red/R), and 940 nm (infrared/IR), penetrating to different layers of skin with irradiating power density of 20 mW/cm^2^ and field of view of 2 × 2 cm^2^. We used the G, R, and IR channels for the quantitative analyses. This device was designed to measure skin diffuse reflectance images by using these four different LED illuminations fixed at 35 mm distance, arranged circularly in the ring and covered by a matt plate diffusor to deliver uniform illumination. Images were collected with a color CMOS 5-megapixel IDS camera (MT9P006STC, IDS uEye UI3581LE-C-HQ, Obersulm, Germany) fixed at 60 mm distance from the illuminated skin [36]. The acquired images were automatically transferred to a cloud server for further data processing and analysis [37]. A long-pass filter (T515 nm > 90%) was inserted in front of the camera, allowing it to capture G, R, and IR spectral channels. The detailed description of this prototype device has been previously published [36,38]. The LED-based multispectral images were analyzed with ImageJ v1.46 software (NIH, Bethesda, MD, USA) [39]. For the intensity analysis and shape description, we manually selected the regions of interests (ROI) using freehand selections. ROIs of melanomas were recorded using the ROI manager function of the ImageJ software. Therefore, the analyzed area was identical in all channels (G, R, IR). We measured the mean gray value (integrated density/area), circularity (4πarea/perimeter^2^), solidity (area/convex area), and roundness (4 × area/(π × major_axis^2^)). 

### 2.4. Differentiation of Nevi from Melanomas with the Use of Parameter s’

We used a novel parameter to exclude nevi as the first step of the melanoma classification algorithm. *Parameter s*’ is based on our previous studies [40,41,42,43].

(1) $$parameter\;s’=\mathrm{lg}\frac{I_{G}\cdot I_{R_{\_skin}}^{2}\;}{I_{G\_skin}\cdot I_{R}^{2}}$$

where *I_G_*: intensity of lesion in green channel, 

*I_G___skin_*: mean intensity of skin in green channel,

*I_R_*: intensity of lesion in red channel,

*I_R_skin_*: mean intensity of skin in red channel. 

We used the patient data of the melanoma patients mentioned before from which 98 patients met the requirement of the parameter s’ assessment (126 image sets). The acquired images were automatically transferred to a cloud server for further data processing and analysis [37]. The image processing algorithms were developed in Matlab (MathWorks) [41]. We used the patient data of the Department of Dermatology, Venereology and Dermatooncology, Semmelweis University (Budapest, Hungary) and the Oncology Center of Latvia (Riga, Latvia), including 143 nevi. 

### 2.5. Melanoma Classification Algorithm

We developed an algorithm to classify melanomas into three subgroups (Breslow tumor thickness ≤ 1 mm, Breslow tumor thickness 1–2 mm, and Breslow tumor thickness > 2 mm) based on the shape descriptors and intensity values of their MSI images. As a first step, this method confirms melanomas and rules out nevi from the calculation. This is followed by the second step where the algorithm carries out the thresholding between low and high circularity (threshold: 0.727; arbitrary unit, A.U.) The next step is the intensity analysis of these two subgroups. Melanomas with low circularity go through an intensity measurement of the G channel (threshold: 8.0 A.U.). Melanomas with a higher G intensity are classified as melanomas with Breslow ≤ 1 mm, and melanomas with lower G intensity are classified as tumors with Breslow 1–2 mm. The IR channel was analyzed for melanomas with low circularity. Melanomas with lower intensities (threshold 107.9 A.U.) were classified as Breslow > 2 mm and melanomas with higher IR intensities were classified as Breslow 1–2 mm.

### 2.6. Dermoscopic Image Analysis by Dermatologists and Dermatology Residents

A spreadsheet-based evaluation form was sent to the dermatologists and dermatology residents of the Department of Dermatology, Venereology and Dermatooncology. This spreadsheet contained dermoscopic images of the 100 investigated melanomas (one to three), a clinical image of the melanomas, and the answers included for the three subgroups (Breslow ≤ 1 mm, Breslow 1–2 mm, Breslow > 2 mm). 

### 2.7. Statistical Analysis

One-way ANOVA was used for statistical analysis to compare the intensity values and shape descriptors. We used receiver operating characteristic (ROC) curves to count the area under the curves (AUC) with default settings (Wilson/Brown method with a confidence interval of 95%). We used Pearson correlation to correlate intensity values with Breslow thickness. Cohen’s kappa (κ) was used to calculate concordance. Statistical tests were performed using GraphPad Prism v8.0.1. software (GraphPad Software Inc., La Jolla, CA, USA) and R version 3.6.1. *p* values below 0.05 were considered statistically significant. The results are expressed as mean ± standard error.

## 3. Results

### 3.1. Patient Data and Histology

**Figure 1:**
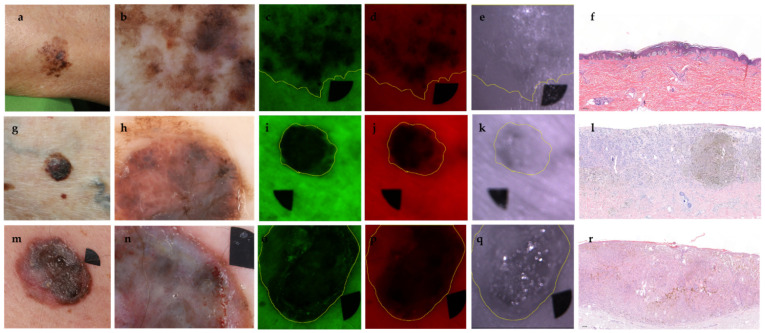
Representative images of melanomas with different Breslow thicknesses. Upper row (**a**–**f**); melanoma with Breslow 0.345 mm, pT1a, Clark II, superficial spreading melanoma (SSM), clinical photograph (**a**), dermoscopic image (**b**), G (**c**), R (**d**), and IR (**e**) channels and histological image (**f**). Middle row (**g**–**l**); melanoma with Breslow 1.81 mm, pT2a, Clark IV, SSM, clinical photograph (**g**), dermoscopic image (**h**), G (**i**), R (**j**), and IR (**k**) channel, histological image (**l**). Lower row (**m**–**r**); melanoma with Breslow 2.42 mm, pT3b, Clark IV, SSM with a nodular component, clinical photograph (**m**), dermoscopic image (**n**), G (**o**), R (**p**), and IR (**q**) channels and histological image (**r**). Black markers (area: 0.125 cm^2^) are used for image alignment. Histology magnification 51× (**f**) and 50× (**l**,**r**) (H&E staining).

In this study, we examined 100 patients with primary melanoma. In total, 128 image sets were collected. Of the 100 melanomas, 69 were SSM (69%), 19 NM (19%), 2 ALM (2%), 3 LMM (3%), 1 naevoid (1%), and 6 unclassified (6%). The mean age of affected patients was 62.64 ± 14.29 years. The sex ratio was 37% women and 63% men. The mean Breslow thickness was 1.777 ± 1.728 mm, ranging from 0.12 mm to 7.5 mm (Figure 1). 

### 3.2. Intensity Values

**Figure 2:**
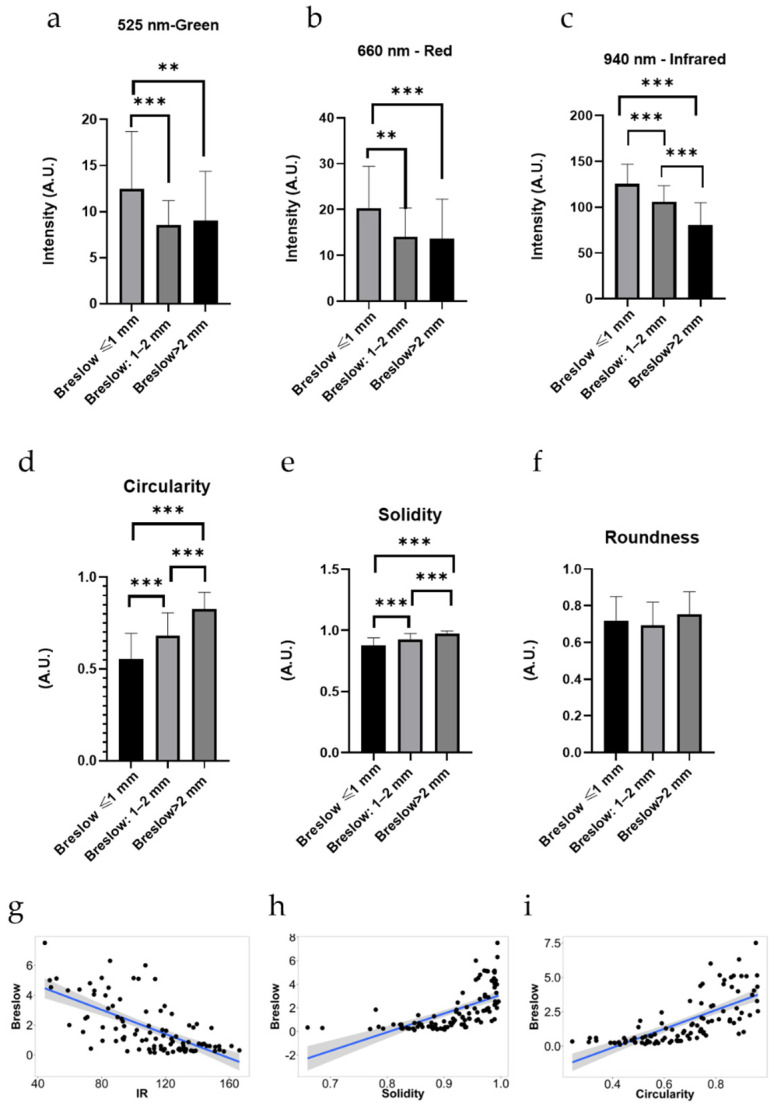
Comparison of melanomas with different tumor thicknesses and correlations between intensity values, shape descriptors, and Breslow thickness. One-way ANOVA and Tukey’s post comparison test were used to compare the intensity values and shape descriptors. The intensity values proved to be statistically significant (**a**) G (*p* < 0.0001), (**b**) R (*p* < 0.0001), and (**c**) IR (*p* < 0.0001) and among the shape descriptors. (**d**) Circularity (*p* < 0.0001) and (**e**) solidity (*p* < 0.0001) were statistically significant. The roundness (**f**) could not separate the three groups effectively (*p*: 0.2759). Moreover, the (G (**a**), R (**b**), and IR (**c**) channels proved to be effective to identify tumors of Breslow ≤ 1 mm from the other two groups, whereas the IR channel could differentiate the Breslow 1–2 mm and Breslow > 2 mm from each other. Pearson’s correlation was used to correlate Breslow thickness with IR, circularity, and solidity. It showed a high correlation between IR intensity (**g**) and Breslow tumor thickness (r: −0.659, 95% confidence interval: −0.7576 to −0.5317, *p*: <0.0001), whereas the correlations between Breslow tumor thickness and G or R intensities were low (r: −0.226 and −0.244, respectively). The correlation was high between solidity and Breslow thickness (**h**) (r: 0.6324 95% confidence interval: 0.4978 to 0.7372, *p*: <0.0001) and high between circularity and Breslow thickness (**i**) (r: 0.7109, 95% confidence interval: 0.5980 to 0.7961, *p*: <0.0001) *p* values between 0.01 and 0.001 were considered very significant (**) and values between 0.001 and 0.0001 were considered extremely significant (***). The results are expressed as mean ± standard error (*n* = 100). A.U. = arbitrary unit.

When the intensity values of various melanomas were studied, we found significant differences in the green (G) and red (R) MSI channels that allowed us to efficiently differentiate the Breslow ≤ 1 mm group from the other two groups. In these tumors, the intensity measured in these channels of Breslow ≤ 1 mm melanomas were significantly higher than in the other two subgroups. Readouts in the infrared (IR) channel did not provide valuable information to distinguish between thinner or thicker, more invasive melanomas. The strongest correlation was between IR intensity and Breslow thickness (r: −0.6593, *p* value: <0.0001, 95% confidence interval: −0.7576 to −0.5317), whereas the G and R channels displayed a lower correlation with tumor thickness (Figure 2).

### 3.3. Shape Descriptors

Among the shape descriptors, both circularity and solidity proved significantly lower in the Breslow ≤ 1 mm group than in the other two subgroups. Investigations of the circularity and solidity made it possible to distinguish the Breslow 1–2 mm and the Breslow > 2 mm subgroups. This was based on the fact that Breslow > 2 mm melanomas had significantly higher circularity and solidity values. However, the roundness did not show any significant differences. Circularity (*p*: <0.0001) and solidity (*p*: <0.0001) proved to be efficient in differentiating thick or nodular melanomas from thin melanomas. Pearson’s correlation showed a high correlation between solidity (r: 0.6324, 95% confidence interval: 0.4978 to 0.7372, *p*: <0.0001) and between circularity and Breslow thickness (r: 0.7109 95% confidence interval: 0.5980 to 0.7961, *p*: <0.0001), whereas the roundness showed no significant differences between the three subgroups (*p* value = 0.2139). 

### 3.4. Differentiation of Nevi from Melanomas with the Use of Parameter s’

**Figure 3:**
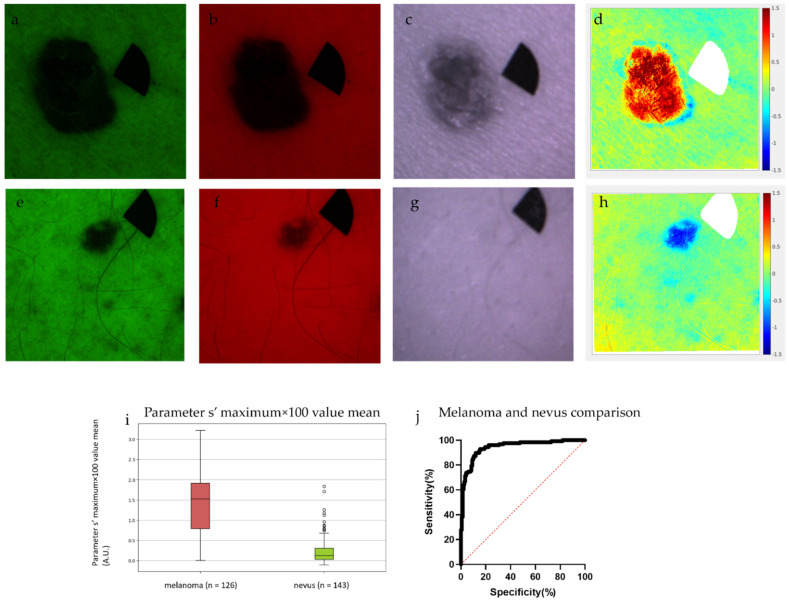
Differentiation of nevi from melanomas with the use of parameter s’. A superficial spreading melanoma, Breslow: 1.02, Clark: IV, pT2a (upper row), and a pigmented nevus (middle row). The parameter maps (**d**,**h**) were calculated using the G (**a**,**e**), R (**b**,**f**), and IR (**c**,**g**) channels. The melanoma with higher parameter s’ is red (**d**), whereas the nevus with its lower parameter s’ is visualized deep blue (**h**). The redness means higher probability of melanoma (highest parameter s’ is 1.5: rufous color), while blueness refers to a higher probability of nevus (lowest parameter s’ is −1.5: deep blue color). Using the maximum values (**i**) with a threshold of 0.511 arbitrary unit (A.U.) melanomas could be differentiated from nevi with a sensitivity of 89.60% and specificity 88.11%. The area under the curve (AUC) was 0.944 (patients: melanoma, control: nevi, 95% confidence interval, *p* < 0.0001) (**j**). *Y*-axis: sensitivity, *x*-axis: 1-specificity.

Parameter s’ was able to distinguish melanomas from nevi with a sensitivity of 89.60% and specificity of 88.11% as the first step of the algorithm. The melanomas had significantly higher parameter s’ values compared to the nevi. The ROC AUC analysis showed significant differences also. The comparison of melanoma and nevus groups had an AUC of 0.944 (patients: melanoma, control: nevi, 95% confidence interval, *p* < 0.0001) (Figure 3). 

### 3.5. Melanoma Classification Algorithm 

**Figure 4:**
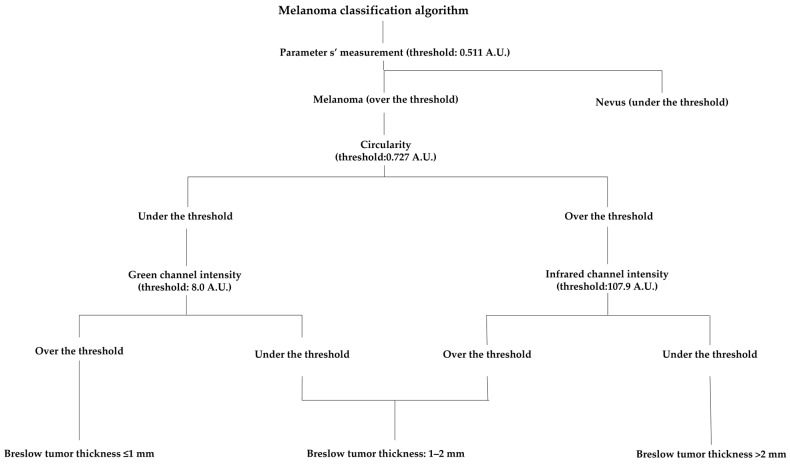
Melanoma classification algorithm. Based on the shape descriptors and intensity values, our melanoma classification algorithm was calculated to classify the multispectral images of melanomas with different Breslow tumor thicknesses. As a first step, this algorithm excludes nevi from the analysis with the use of parameter s’ (threshold: 0.511 A.U.). The second step is a threshold between lower and higher circularities that was established (threshold: 0.727 A.U.), which sorted melanomas into two groups: (1) low and (2) high circularity. The third step was the classification of melanomas from these two subgroups to the three previously defined groups (Breslow tumor thickness ≤ 1 mm, Breslow tumor thickness 1–2 mm, Breslow tumor thickness > 2 mm). We used the intensity values of green channel (threshold: 8.0 A.U.) and the infrared channel (threshold: 107.9 A.U.). This algorithm was able to classify melanomas into three subgroups with a sensitivity of 78% and specificity of 89%.

We have developed a novel melanoma classification algorithm based on MSI shape descriptors and intensity values that allow us to classify melanomas into the above-mentioned three subgroups with a sensitivity of 78% and specificity of 89%, (Figure 4). The sensitivities for each subgroup were 80.85% (Breslow ≤ 1 mm), 76.19% (Breslow 1–2 mm), and 81.25% (Breslow > 2 mm). The specificities were 96.22% (Breslow ≤ 1 mm), 82.27% (Breslow 1–2 mm), and 94.11% (Breslow > 2 mm). The total agreement for predicting the right subgroup was substantial (κ = 0.67; 95% CI, 0.58–0.76), also it was substantial classifying melanomas to Breslow ≤ 1 mm subgroup (κ = 0.76; 95% CI, 0.63 to 0.89) and to Breslow > 2 mm subgroup (κ = 0.73; 95% CI, 0.59 to 0.88). The agreement was moderate when the algorithm classified melanomas to the Breslow 1–2 mm subgroup (κ = 0.47; 95% CI, 0.28 to 0.65). 

### 3.6. Dermoscopic Image Analysis by Dermatologists and Dermatology Residents

The total sensitivity of their categorization into the three groups described above was 60.38%, while the specificity was 80.86% with a moderate total agreement (κ = 0.41; 95% CI, 0.40 to 0.43) (Table 1). The sensitivity of the assessment by dermatologists was 62.19% with a specificity of 81.09% and a moderate agreement (κ = 0.44; 95% CI, 0.42 to 0.47), whereas the sensitivity of the evaluation by dermatology residents was 58.44%, with a specificity of 79.76% and a fair agreement (κ = 0.39; 95% CI, From 0.36 to 0.41). Among subgroups, classifying into the Breslow > 2 mm subgroup had the highest total sensitivity of 90.37% and specificity of 78.58% with high substantial agreement (κ = 0.65; 95% CI, 0.61 to 0.69). Classification into the Breslow ≤ 1 mm subgroup had a sensitivity of 51.69% and specificity of 96.95% with a moderate agreement (κ = 0.49; 95% CI, From 0.46 to 0.52). The classification into the Breslow 1–2 mm subgroup had a sensitivity of 38.51% and specificity of 72.07% with no agreement (κ = 0.09; 95% CI, 0.06 to 0.13).

## 4. Discussion

MSI allows the examiner to use several wavelength-dependent features and has been previously used to detect melanomas. However, these studies mainly focused on the differentiation of melanomas from other skin lesions [44,45,46,47,48,49], and only a few studies focused on depth prediction [50]. To the best of our knowledge, we were the first to analyze melanoma tumor thickness with multispectral imaging to classify melanomas into subgroups of great clinical relevance. This was all based on the analysis of their shape descriptors and intensity values. Shape descriptors efficiently differentiated high and low-risk melanomas, namely over 2 mm vs. less than 1 mm. SMMs are more common among thin melanomas (*p* < 0.001) and NMs are more common among patients with thick melanomas (*p* < 0.001) [51].The combination of shape descriptors and intensity values was sensitive and specific enough for the melanoma classification algorithm to sort melanomas into the three categories, with a sensitivity of 78.00%, specificity of 89.00% with a substantial agreement (κ = 0.67; 95% CI, 0.58–0.76). Circularity, the sphericity of lesions was the most suitable shape descriptor to classify melanomas into a low- and high-risk group as the second step. Larger and thicker melanomas were more circular. However, circularity alone is not sufficient to accurately establish melanoma thickness, thus a third classification step was needed. 

The third analysis step relied on analysis more closely related to the dermal localization of melanoma cells. These findings are in line with our previous findings as shorter wavelengths, such as G and R, penetrate the dermis only superficially and are absorbed and reflected by tumor chromophores mainly from the surface [33]. The IR penetrates deeper to the skin and is reflected by tumor chromophores deeper from the dermis [52], consistently with the literature [53]. Therefore, G channel was suitable to differentiate between Breslow ≤ 1 mm melanomas and Breslow 1–2 mm melanomas, whereas the IR channel could distinguish between Breslow 1–2 mm melanomas and melanomas with higher than 2 mm Breslow thickness. Because of its physical characteristics, the G channel was more useful to identify superficial lesions. Thinner melanomas had higher intensities because of the lower melanin concentration, whereas G channel could not differentiate between Breslow 1–2 mm and Breslow > 2 mm melanomas. The IR channel was able to provide information about the deeper layers of the skin. Therefore, it was effective to distinguish better between Breslow 1–2 mm and Breslow > 2 mm melanomas. Thinner melanomas are characterized by a higher chance of regression and presence of hypopigmented areas [16], which lesions had higher intensity values in both G and IR channels. 

We built in an additional first step into the algorithm to exclude nevi from the analysis using parameter s’. Parameter s’ is an improved formula based on our previous findings to differentiate melanoma from nevi [40,41,42,43]. It utilizes the intensity values of the lesion and the surrounding skin in G and R channels to calculate a predictive value. In our study, melanomas had significantly higher parameter s’ values compared to nevi. Therefore, with our thresholding algorithm nevi could be differentiated from melanomas with a sensitivity of 89.60% and specificity of 88.11% (Figure 3). These findings are consistent with the literature, where multispectral imaging had been previously applied successfully to differentiate these two entities [45]. Beyond multispectral imaging, melanoma and nevus differentiation is one of the most researched topics in dermatology using various imaging modalities. Many studies have focused on this problem and used computerized and AI-aided methods to differentiate benign lesions from malignant skin tumors [54,55,56,57]. This is a potentially applicable step to exclude benign pigmented nevi and reconsider the clinical diagnosis when our algorithm is used to estimate tumor thickness of melanoma. 

In this study, we also compared the performance of our MSI-based algorithm to that of human observers. Clinical and dermoscopic images of 100 melanomas were shown to dermatologists and dermatology residents to assess their ability to classify the lesion based on tumor thickness. Dermatologists and dermatology residents completed the form with a total sensitivity of 60.38%, of which the dermatologists reached a sensitivity of 62.19%, and the dermatologist residents performed at a sensitivity rate of 58.44%. Specificity reached 80.86%, with 81.09% and 79.76% for dermatologists and dermatology residents, respectively. The total agreement was found to be moderate (κ = 0.41; 95% CI, 0.40 to 0.43). Compared to the melanoma classification algorithm, all human investigators achieved a lower sensitivity and specificity in classifying melanomas into subgroups based on likely histological tumor thickness. Humans had lower accuracy, and the agreement was higher using the algorithm (κ = 0.67; 95% CI, 0.58–0.76). However, it is important to note that palpation is an important guide to clinicians to aid their vision when estimating the tumor thickness during routine examinations, which was not possible in this study. These data were similar to earlier findings in the literature. Dermoscopy was recently described to predict Breslow tumor thickness with a concordance of 0.52, and it could even differentiate between in situ melanomas and tumors thicker than 1 mm [15].

This MSI technique and our novel algorithm is a potential tool to aid clinicians in the evaluation of melanoma depth. It is fast and easy-to-perform, the imaging takes 20 s whereas running the algorithm needs one minute. It is comparable to other modalities, such as HFUS, which could estimate the required surgical margins of melanomas (1, 2, or 3 cm) in 26 of the 31 subjects [13]. Moreover, preoperative HFUS was found to be a potential tool aid for the excision of melanoma in one step [58]. Combining HFUS with digital dermoscopy enhanced the accuracy also, and could differentiate thick and thin melanomas with a sensitivity of 86.7% [59]. Optical coherence tomography is a potential tool also to predict melanoma tumor thickness based on their vascular morphology [60]. Reflectance confocal microscopy proved to be an accurate modality in the presurgical margin mapping of only LMMs [61,62]. Although these imaging modalities can be used to estimate Breslow tumor thickness, compared to MSI, their main disadvantage is that they are expensive, and their efficacy depends fundamentally on the examiner’s skill and proficiency [63,64,65].

This multispectral LED-based imaging and our algorithm has several limitations. The field of view of 2 × 2 cm^2^ of the device limits the maximum area of the imaging. Another limitation is the acquisition of images of special sites (acral, genital, or mucosal melanoma) is also not possible. The algorithm we created could be potentially further improved in the future. The acquired multispectral images could be applicable as training data for machine learning algorithms. The MSI imaging procedure takes 30 s and an additional 30 s is required to upload the images to the cloud server. It takes around one minute to manually carry out the required image analysis for the algorithm and complete the classification. The automatization of this melanoma classifying algorithm is one of our future plans. As a result, the time required for melanoma classification could be further reduced. The last limitation factor is the fact that a multispectral LED device prototype is not widely available yet and it is difficult to compare our results to MSI findings acquired with other imaging settings. The Breslow thickness and its connection with the progression of melanoma is thoroughly investigated in the literature. Thick melanomas show a high risk of early metastasis and locoregional spread [66]. Kulkarni et al. also noted histologic patterns that predict a higher risk of recurrence based on H&E images processed by deep learning [67]. Breslow thickness and ulceration remain the main predictors of prognosis. Paolino et al analyzed 244 acral melanomas and also noted the importance of these two factors [68]. 

## 5. Conclusions

MSI is a potentially helpful tool to determine the required surgical margin based on the estimated Breslow thickness. It is easy-to-access, cost-effective, and can be used as a mobile add-on device using the camera of a smartphone. The collected data may serve as a training pool for machine learning algorithms for further improvements in order to achieve a more accurate estimation of Breslow thickness, as Marchesini et al. suggested [50].

MSI and dermoscopy are non-invasive imaging modalities. Both can help the specialists estimate melanoma tumor thickness and can aid the differentiation of nevi from melanomas. However, dermoscopy is a tool designed for healthcare professionals with dedicated training [13,51,69]. In contrast, MSI requires no previous training and may be used as smartphones attachment to estimate tumor thickness. Based on our findings, MSI may be used in clinical practice for the prediction of appropriate safety margins for curative melanoma excisions.

## Figures and Tables

**Table 1:** Comparison of the melanoma classification algorithm and the assessment based on dermoscopic and clinical images by dermatologist and dermatology residents (*n* = 100).

	Melanoma Classification Algorithm	Assessment Based on Dermoscopic and Clinical Image
Cohen’s kappa	0.67	0.41
Sensitivity	78.00%	60.38%
Specificity	89.00%	80.86%

## Data Availability

The data that support the findings of this study are available from the corresponding author N.M.W. upon reasonable request.
